# Epigenetic regulation of human embryonic stem cells

**DOI:** 10.3389/fgene.2012.00238

**Published:** 2012-11-05

**Authors:** Qidong Hu, Michael G. Rosenfeld

**Affiliations:** School/Department of Medicine, Howard Hughes Medical Institute, University of CaliforniaSan Diego, CA, USA

**Keywords:** embryonic stem cell, epigenetic regulation, DNA methylation, chromatin remodeling, DNA repeat

## Abstract

Recently, there has been tremendous progress in characterizing the transcriptional network regulating human embryonic stem cells (hESCs; MacArthur etal., 2009; Loh etal., 2011), including those signaling events mediated by *Oct4*, *Nanog*, and *Sox2*. There is growing interest in the epigenetic machinery involved in hESC self-renewal and differentiation. In general, epigenetic regulation includes chromatin reorganization, DNA modification, and histone modification, which are not directly related to alterations in DNA sequences. Various protein complexes, including Polycomb, trithorax, nucleosome remodeling deacetylase, SWI/SNF, and Oct4, have been shown to play critical roles in epigenetic control of hESC physiology. Hence, we will formally review recent advances in unraveling the multifaceted role of epigenetic regulation in hESC self-renewal and induced differentiation, particularly with respect to chromatin remodeling and DNA methylation events. Elucidating the molecular mechanisms underlying the maintenance/differentiation of hESCs and reprogramming of somatic cells will greatly strengthen our capacity to generate various types of cells to treat human diseases.

## CHROMATIN REMODELING IN hESCs

Human embryonic stem cells (hESCs) feature the capability to self-renew and to differentiate into all types of cells in humans. Hence, they are therapeutically invaluable to treat major human diseases, such as neurodegeneration, diabetes, and cardiovascular diseases. Recently, various approaches have been developed to reprogram human somatic cells into induced pluripotent stem cells (iPS cells; Yamanaka, 2009), providing a more abundant and ethical-feasible source of progenitor cells that possess the capacity of generating all types of human cells. However, the low efficiency of reprogramming terminally differentiated cells into iPS cells has remained a major obstacle that prevents the wide application of this technology in practical use. Hence, to achieve a more comprehensive understanding of hESC biology and to promote application of the stem cell strategy in treating devastating diseases, scientists have begun to explore new territories in the context of hESC maintenance/differentiation, including the role of chromatin remodeling.

It is well established that chromatin is composed of the DNA component, the histone components being wrapped and other related proteins. The dynamic assembly or disassembly of DNA-histone structure is closely associated with many important biological processes, such as DNA replication, DNA repair, and transcription. Previous studies have shown that in hESCs, the chromatin is in a less compact state globally than in terminally differentiated cells (Meshorer etal., 2006). This loose compaction of chromatin facilitates more dynamic and flexible reorganization during differentiation.

Many complexes, collectively referred to as chromatin remodelers that are mostly ATP-dependent, have been identified to contribute to this less compact state. Chromatin remodelers mediate the interaction between the DNA helix and histones, hence regulating the accessibility of the DNA to transcription factors and other machinery (Ho and Crabtree, 2010). Some well-characterized remodeling complexes include SWI/SNF, NuRD, Tip60/p400, and chromodomain helicase DNA binding protein (CHD), which will be detailed in the following.

The SWI/SNF complex contains 9–12 subunits, among which BAFs (including BAF47 and BAF155/170) play critical regulatory roles (Phelan etal., 1999) while Brg1 and Brm possess the ATPase activity (de la Serna etal., 2006). Both Brg1 and Brm contain bromodomains, hence they exhibit a preference for acetylated histones. These proteins are ubiquitously utilized in mammalian development, both in the maintenance of stem cell state and in differentiation. In particular, Brg1 has been shown to interact with the key regulators of pluripotency, Oct4, Sox2, and NANOG, and exhibits a highly correlated genome-wide binding patterns with these proteins (Liang etal., 2008; Ho etal., 2009), suggesting a cooperative role of SWI/SNF complexes in keeping the cells in the undifferentiated state. In addition, Brg1 binds to many development-related genes exhibiting bivalent histone marks that represent a “poised” status of activation. The bivalent histone mark includes the H3K4me3 activation and the H3K27me3 repressive modifications, which are particularly confined to embryonic stem cells (Bernstein etal., 2006). Hence Brg1 coverage of these regions may ensure an efficient and reversible repression of differentiation-related genes. Once differentiation is irreversibly initiated, BAF155 increases the H3K27me3 modification at the *NANOG* promoter and consequently causes condensation of the local chromatin, hence repressing the expression of *NANOG* (Schaniel etal., 2009). If BAF155 is depleted, the expression level of *NANOG* will remain high so that differentiation of hESCs will be significantly impaired. Additionally, genome-wide location analysis has found that BAF155 is also involved in depositing on chromatin the H3K9me3 mark, a modification that contributes to heterochromatin formation (Schaniel etal., 2009). Hence, even though co-existed in one SWI/SNF complex, Brg1 and BAF155 exert somewhat opposing effects on stem cell maintenance and differentiation.

The Mi-2/nucleosome remodeling deacetylase (NuRD) complex possesses both the APT-dependent chromatin remodeling activity and the histone deacetylase activity (Denslow and Wade, 2007). The key components include chromodomain helicase CHD3/4, deacetylase HDAC1/2, methy-CpG-binding proteins Mbd3 and Mta1. They are involved in regulating pluripotency and differentiation of ESCs via histone deacetylation. The deficiency of Mbd3 leads to hyperacetylation and loss of ES pluripotency (Zhu etal., 2009). For a similar complex that lacks Mbd3, NODE, studies have found that it interacts with NANOG and Oct4, and co-binds to the NANOG/Oct4 target genes (Liang etal., 2008). Depletion of Mta1 de-represses genes related to endoderm differentiation, such as GATA6 and FoxA2 (Liang etal., 2008), indicating that an intact NODE complex is required for suppression of premature cell lineage commitment.

The Tip60/p400 complex also contains both an ATPase activity and an acetyltransferase activity. In particular, the ATPase activity is conferred by p400. Both activities are required for early embryonic development (Fazzio etal., 2008; Hu etal., 2009). Depletion of Tip60/p400 leads to de-repression of genes, many of which are important developmental regulators. Down-regulation of this complex also impairs self-renewal of progenitor cells. Consistently, p400 binding profile coincides with those of H3K4me3 and bivalent marks, a unique epigenetic feature in ESCs (Fazzio etal., 2008), implying that the Tip60/p400 complex is involved in maintaining the pluripotent state of the cell.

A fourth major remodeling family is the CHDs. They contain two chromodomains, hence exhibiting high affinity for methylated histones, especially H3K4me2/3 (Flanagan etal., 2005; Marfella and Imbalzano, 2007; Sims etal., 2007). One member of particular significance is CHD1, which has been proved to be required for maintaining a loose/open chromatin conformation in ESCs (Gaspar-Maia etal., 2009). Depletion of CHD1 leads to heterochromatin formation characterized by high enrichment of H3K9me3 and HP1γ, down-regulation of Oct4, and initiation of neural development (Gaspar-Maia etal., 2009). Another major member, CHD7, has been found critical for generation of migration-competent neural crest-like cell from hESCs (Bajpai etal., 2010). In humans, mutation of CHD7 can also cause a genetic disorder, CHARGE, which is characterized by severe defects in many cell types at birth (Vissers etal., 2004), implying that CHD7 is indeed involved in embryonic development. It is interesting to note that CHD7 harbors the BRK domain, which is known to bind to CTCF (Allen etal., 2007), the protein playing as insulators, hence raising the possibility that CHD7 may help create a chromatin landscape with active (self-renewal and pluripotency-related genes) and quiescent (cell lineage-specific genes) segments.

## DNA METHYLATION REGULATION IN hESC MAINTENANCE AND DIFFERENTIATION

DNA methylation mainly occurs at the 5-C position of the CG dinucleotide in mammalian cells, the occurrence of which is inversely correlated with the GC content and CpG density (Bird, 2002; Illingworth and Bird, 2009). It represents a major epigenetic regulation for many biological processes, such as gene transcription, imprinting and transposon activity in embryonic stem cells, germ cells, somatic cells, and tumor cells (Aranyi and Paldi, 2006; Farthing etal., 2008; Xie etal., 2009).

DNA methylation is accomplished by three independent DNA methyltransferases, DNMT1, DNMT3A, and DNMT3B (Kato etal., 2007). In particular, DNMT3A and 3B are responsible for *de novo* methylation events at cysteine while DNMT1 is involved in the maintenance of the methylated status (Okano etal., 1999; Chen etal., 2003). Depletion of DNMT members leads to embryonic lethality in the mouse model (Li etal., 1992; Okano etal., 1998), and in *in vitro* culture, even though *DNMT*-knockout cells can still self-renew, they lose the potentiality to differentiate (Thomson etal., 1998; Okano etal., 1999), suggesting that DNMT plays a more prominent role in pluripotency. DNA methylation plays a critical epigenetic role in terms of gene expression (Loh etal., 2011) and has different patterns in hESCs and differentiated cells. In hESCs, the whole genome is relatively hypomethylated, reflecting a more open and hyperdynamic nature of chromatin, while in the latter, there is more DNA methylation genome-wide suggesting a highly organized and a less active chromatin conformation (Robertson, 2001; Bibikova etal., 2006; Gan etal., 2007). Previous research has revealed an interesting inverse correlation between CpG methylation and CpG dinucleotide density, whereby densely packed CpG islands appear to be protected from methylation (Kafri etal., 1992). Generally, CpG islands near promoters of actively expressed genes are demethylated and methylation at these sites is typically associated with gene repression during development (Bird, 2002). However, methylation within the gene is usually associated with active transcription of the gene, indicating that DNA methylation at different regions of genes can play opposite roles (Laurent etal., 2010). In hESCs, the CpG islands in the promoters of housekeeping genes and key stem cell signature genes, such as *Oct4*, *NANOG*, *TDGF1*, and *Sox2*, remain demethylated, hence permitting their active expression to maintain the stem cell state (Weber etal., 2007; Meissner etal., 2008). This notion is strengthened by studies using the DNA methyltransferase inhibitors, 5-azacytidine and RG108, in which cases the efficiency of reprogramming mouse fibroblasts to iPS cells were greatly enhanced (Mikkelsen etal., 2008; Shi etal., 2008).

In a recent study, a substantial amount of non-CpG methylation was also uncovered in hESCs (Lister etal., 2009). However, these non-conventional methylation simply disappeared upon induced differentiation, indicating that ESCs uniquely employ an additional methylation strategy to epigenetically regulate gene expression. By pairwise comparison of hESC and fetal fibroblasts, the same group also observed a differentiation-related decrease in CpG methylation as well as a positive correlation between CpG methylation level in the gene body and gene expression in differentiated cells.

In addition, DNA methylation occurred in repetitive sequences has started to draw increasing attention from the field, partially due to the fact that in human cells, gene-coding regions only takes up ~2% of the whole genome (Lander etal., 2001), while ~47% of the DNA content can be classified into different types of repetitive elements – long-interspersed element (LINE), short-interspersed element (SINE), SVA, LTR, satellite repeats, etc. A typical LINE repeat occupies a ~6 kb DNA sequence. In the human genome, LINE repeats are mainly located outside coding sequences of genes, such as promoters, introns, and untranslated regions. In hESCs, they are constitutively transcribed into RNAs. Two open reading frames, ORF1 and ORF2 (Scott etal., 1987), cannot only be transcribed into RNAs, but also produce retrotranscriptase that is essential for transposition of other retrotransposons, such as Alu repeats. Additionally, ORF1 is transcribed and translated into a protein with RNA binding and chaperon activity (Hohjoh and Singer, 1997; Martin and Bushman, 2001). Alu repeats are evolved from the 7SL RNA, and are typically 300 bp in length. There are about one million copies of Alu repeats which account for ~11% DNA contents of the human genome (Rubin etal., 1994). They are scattered within both coding and non-coding regions. In general, these repetitive DNA elements had once been referred to as “junk DNA” since no definitive functions had been uncovered for them. However, recent work has suggested that they represent an overlooked “treasure chest” with huge regulatory potentiality to exert tremendous influence on cells, including the integrity of human genome, embryogenesis, and tumorigenesis.

Significantly, over half of CpG methylation events in the human genome is distributed among various types of DNA repeats (Cordaux and Batzer, 2009), but the methylation density varies greatly among different classes of DNA repeats. In particular, about one-third of the CpG islands are embedded in Alu repeats which exhibit CpG density-correlated methylation, whereas CpG islands embedded in LINE and LTR elements are generally hypermethylated regardless of the GC content (Meissner etal., 2008).

Hypermethylation contributes to silencing of these repeats and formation of heterochromatin. Since LINE, Alu and SVA repetitive elements are frequently located in proximity to genes with protein coding capacities (Cordaux and Batzer, 2009), these inhibitory effects will most likely spread to the adjacent genes, thus epigenetically regulating gene expression in hESCs. Additionally, in terminally differentiated human cells, repetitive elements are generally highly methylated and the quiescence of these repeats is required for the integrity and stability of the cell genome (Donnelly etal., 1999; Kato etal., 2007).

Recent studies have found that many repetitive elements are hypermethylated in human pluripotent cells as compared to differentiated cells (Meissner etal., 2008; Su etal., 2012), suggesting an active role of these elements in maintaining the stem cell state. The genome-wide analysis has shown that cells experience dramatic changes in DNA methylation pattern during cell fate commitment (Meissner etal., 2008; Lister etal., 2009; Nagae etal., 2011). In particular, LINE, LTR, and satellite elements show a more significant differentiation-induced decrease in CpG methylation than SINE, RNA repeats, and SVA repeats (Su etal., 2012). This differential demethylation pattern can be partly explained by the relative lower CpG density along the DNA sequences of LINE and LTR (Su etal., 2012). Their study has further found that the demethylated repetitive elements are mainly distributed in intergenic regulatory regions and introns, but not in coding exons (Meissner etal., 2008; Su etal., 2012), suggesting a potential epigenetic regulation of the adjacent coding region. Indeed, genomic profiling of the demethylated repetitive elements has shown that most of the intron-embedded repeats are methylated in hESCs but demethylated in differentiated cells. The genes containing demethylated repetitive elements in their introns generally exhibit a significantly lower level of expression, suggesting a synergized regulation of methylation in gene-embedded repeats and gene transcription (Ball etal., 2009; Su etal., 2012). Interestingly, in many human cancers, CpG hypomethylation has been observed in repetitive DNA elements, LINE, SINE, LTR, and satellite repeats (Rauch etal., 2008; Bollati etal., 2009; Choi etal., 2009; Igarashi etal., 2010; Xie etal., 2010), reflecting that tumorigenesis potentially shares some common epigenetic regulation pathways with hESC maintenance and somatic cell reprogramming.

## CROSSTALK BETWEEN HISTONE MODIFICATION AND DNA METHYLATION

Histone modifications represent another major field of research on epigenetic regulation of gene expression, and there are many excellent reviews recently published (Kato etal., 2011; Loh etal., 2011). Hence, in the paper, we instead focus on the crosstalk between DNA methylation and histone modification.

Studies have identified an inverse relationship between H3K4 methylation and DNA methylation (Hashimshony etal., 2003; Meissner etal., 2008). One study observed that methylation at CpG-dense regions will inhibit the local Trithorex activity that confers H3K4 methylation modification (Bird, 2002). Since H3K4me3 is usually associated with gene activation and present at CpG islands largely enriched in promoter regions, these mutually exclusive effects may be part of the coordinative mechanism underlying gene activation and silencing.

At the protein level, an interaction between methylcytosine binding proteins, MeCP2/Mbd2, and HDAC/HMT has been observed (Jones etal., 1998; Nan etal., 1998; Fuks etal., 2003). However, a causal relationship has not yet been established. There are also reports indicating that G9a and ESET, two methyltransferases for H3K9, can interact with DNMT3A/3B to facilitate local methylation of DNA (Feldman etal., 2006; Li etal., 2006). EZH2, an enzymatic component of the PRC2 complex involved in H3K27 methylation, can also interact with DNMTs to influence the DNA methylation status (Viré et al., 2006).

## CONCLUDING REMARKS

Recent studies on epigenetic landscape of embryonic stem cells have gradually unraveled another layer of regulation on pluripotency maintenance and differentiation. However, our current knowledge of the spatiotemporal alterations in epigenetic modifications that accompany ESC maintenance and transition/differentiation into functional somatic cells has remained very limited, which prevents us from drawing a comprehensive image of epigenetic regulatory network in hESCs. The crosstalk between different epigenetic machinery, chromatin remodeling, histone modifications, and DNA methylation, certainly warrants future explorations to elucidate their respective roles in the epigenetic regulation. We are also facing the challenge of integrating the transcriptional regulation network and epigenetic network to fully understand hESC self-renewal and differentiation. Furthermore, another major component of the central theorem, RNA, is gradually coming into sight regarding the pluripotency maintenance and development. Recent studies on non-coding RNAs and small RNAs have suggested that some RNA species could regulate CpG methylation at promoter regions and gene expression by interacting with DNMT (Cernilogar etal., 2011; Rajasethupathy etal., 2012), adding another twist to the already exciting research field of epigenetic regulation in hESCs.

## Conflict of Interest Statement

The authors declare that the research was conducted in the absence of any commercial or financial relationships that could be construed as a potential conflict of interest.
